# PREGABALIN AS A PREOPERATIVE ADJUVANT IN PATIENTS WITH CARPAL TUNNEL SYNDROME

**DOI:** 10.1590/1413-785220243205e278892

**Published:** 2024-10-28

**Authors:** Fábio Hideki Nishi Eto, Thiago Broggin Dutra Rodrigues, Victor Elzio Gasperoni Matias, Yussef Ali Abdouni

**Affiliations:** 1. Irmandade Santa Casa de Misericórdia de São Paulo, Departamento de Ortopedia e Traumatologia “Pavilhao Fernandinho Simonsen”, Grupo de Mão e Microcirurgia, Sao Paulo, SP, Brazil.; 2. Irmandade Santa Casa de Misericordia de São Paulo, Departamento de Ortopedia e Traumatologia “Pavilhão Fernandinho Simonsen”, Sao Paulo, SP, Brazil.

**Keywords:** Carpal Tunnel Syndrome, Surgical Procedures, Operative, Pregabalin, Pain, Síndrome do Túnel do Carpo, Procedimento Cirúrgico, Pregabalina, Dor

## Abstract

Objective: To evaluate the pregabalins adjuvant effect in patients with carpal tunnel syndrome (CTS) surgically treated, analyzing postoperative pain and the incidence of complex regional pain syndrome (CRPS). Methods: Outpatient surgical candidates with CTS were selected and followed for 12 months, divided into three groups. The Control Group received a placebo, the Pregabalin 75 mg Group received a daily dose, and the Pregabalin 150 mg Group received a daily dose of the medication. Patient progress was evaluated using the visual analog scale (VAS) for pain and the DN4 neuropathic pain score before surgery, one month and three months after. Results: The administration of pregabalin to surgical patients with CTS did not demonstrate significant differences in immediate postoperative pain relief. Additionally, there were no statistically significant variations in the incidence of complications, such as CRPS, among the groups. Conclusion: This study did not show a significant impact of pregabalin on postoperative pain relief or the reduction of CRPS incidence in patients undergoing surgery for CTS. These results suggest that pregabalin might not be an effective adjuvant in these surgical situations. ***Level of Evidence II, Comparative prospective study*** .

## RESUMO

Objetivo: Avaliar o efeito adjuvante da pregabalina em pacientes com síndrome do túnel do carpo (STC) tratados cirurgicamente, analisando a dor pós-operatória e a incidência da síndrome da dor complexa regional (SDCR). Métodos: Foram selecionados pacientes com acompanhamento ambulatorial, indicação de tratamento cirúrgico para STC e acompanhados ao longo de 12 meses. Depois, foram divididos em três grupos. O Grupo Controle recebeu placebo, o Grupo Pregabalina 75 mg tomou uma dose diária e o Grupo Pregabalina 150 mg também recebeu dose diária da medicação. A evolução dos pacientes foi avaliada mediante aplicação da escala visual analógica de dor (EVA) e o escore de dor neuropática DN4, antes da cirurgia, um mês e três meses após. Resultados: A administração de pregabalina em pacientes cirúrgicos com STC não demonstrou diferenças significativas no alívio da dor pós-operatória imediata. Além disso, não houve variações estatisticamente significativas na incidência de complicações, como a SDCR, entre os grupos. Conclusão: Este estudo não evidenciou um impacto significativo da pregabalina no alívio da dor pós-operatória ou na redução da incidência da SDCR em pacientes submetidos a cirurgia para STC. Esses resultados sugerem que a pregabalina pode não ser um adjuvante eficaz nessas situações cirúrgicas. ***Nível de Evidência II, Estudo prospectivo comparativo*** .

## INTRODUCTION

 Carpal tunnel syndrome (CTS) is the most common compression neuropathy of the upper limbs and affects approximately 4% of the general population, with a higher prevalence in women aged 45 to 60 years. ^1^ The clinical picture is characterized by pain and paraesthesia in the median nerve area with insidious onset and, in more severe cases, muscle weakness and atrophy of the thenar muscles. ^2^ The carpal tunnel is an osteofibrous, inelastic canal with the transverse carpal ligament as its roof, through which 10 structures pass, including nine tendons and the median nerve. 

 Surgical treatment of CTS involves release of the transverse ligament and decompression of the nerve. ^3^ Although it is a common procedure in hand surgery with high success rates, CTS surgery can have unsatisfactory outcomes for patients, including complications that cannot be prevented, such as the development of chronic postoperative pain. ^4^

 Complex regional pain syndrome (CRPS), also known as reflex sympathetic dystrophy or causalgia, is a chronic pain disorder with neuropathic features that are often out of proportion to the nociceptive stimulus and may or may not be accompanied by vasomotor changes. ^5^ It is more common in women and prolonged immobilization may act as a predisposing factor. The incidence of CRPS after carpal tunnel release surgery is about 8% ^6^ , regardless of technique, and accounts for half of the complications after this type of surgery in some case series ^7^ . 

 With the understanding of the central sensitization processes that lead to chronic pain, drugs from the gabapentinoid class, particularly pregabalin, have been investigated for the prevention of CRPS. Most studies in the literature analyzed the reduction in pain scores and opioid consumption in knee surgery when pregabalin was used preventively. ^8^ ^,^ ^9^ However, there is still no consensus on the dose or duration of use of these drugs. This study aims to evaluate the efficacy of pregabalin as an adjuvant in the preoperative phase of carpal tunnel release surgery. 

## MATERIALS AND METHODS

In this study, patients presenting to the hand surgery outpatient clinic of Santa Casa de Misericórdia de São Paulo with a diagnosis of CTS between June 2022 and June 2023 were analyzed and prospectively observed. The study was conducted with the approval of the Research Ethics Committee of the same institution, according to Resolution 196/96 (CAAE: 69653223.9.0000.5479), and all patients signed the informed consent form (ICF).

These patients were randomized into three groups:

- Patients who received placebo in the three weeks before surgery.
- Patients who received 75 mg/day of pregabalin in the three weeks before surgery.
- Patients who received 150 mg/day of pregabalin in the three weeks before surgery.

Patients of both sexes aged between 40 and 70 years who had a confirmed diagnosis of CTS by ultrasound, electromyography or clinical examination participated in the study. Surgical decompression of the median nerve was performed by the same surgeon using the mini-incision technique. Patients with previous surgery on the same hand, concomitant neuropathies, previous use of gabapentinoids and a history of CRPS were excluded from the study.

Data were collected and analyzed by the same researcher using the visual analog pain scale (VAS) and the DN4 neuropathic pain questionnaire. All patients followed the same follow-up protocol. Medication or placebo was started three weeks before the procedure, and examinations were carried out immediately after the procedure and one month and three months after the procedure. All patients wore an orthosis for immobilization until the stitches were removed, which took place two weeks after the procedure. They also received simple analgesia with NSAIDs and tramadol for pain relief for the first two weeks if needed. They were also supervised by the occupational therapy team until the last examination.

 Patients were examined before the procedure, one month and three months postoperatively. Quantitative characteristics were described by group using summary measures (means, standard deviations, medians and quartiles) and compared between groups using analysis of variance (ANOVA) or the Kruskal-Wallis test. Qualitative characteristics were described by the group using absolute and relative frequencies and tested for association using the likelihood ratio test. ^10^

 Pain scores were described by the group across evaluation periods using summary measures and compared between groups and periods using generalized estimating equations (GEE) with normal marginal distribution and identity link function assuming a first-order autoregressive correlation matrix (AR(1)) between evaluation periods. ^11^ The analyses were followed by Bonferroni ^12^ multiple comparisons to test for differences between groups and periods. 

Analyses were performed using IBM-SPSS for Windows version 22.0, tabulated using Microsoft Excel 2013, and tests were performed at a 5% significance level.

## RESULTS

Among the 45 patients diagnosed with CTS and indicated for surgical treatment, 18 patients were in group 1 (placebo in the three weeks before surgery), 15 patients were in group 2 (75 mg/day pregabalin in the three weeks before surgery), and 13 patients were in group 3 (150 mg/day pregabalin in the three weeks before surgery). Four patients were excluded due to prior use of gabapentin, loss to postoperative follow-up, and noncompliance with suggested medication before surgery.

 Both the VAS and DN4 showed statistically similar mean behavior across the assessment periods (interaction p > 0.05). The VAS showed differences between groups regardless of assessment period (group p = 0.007) and the VAS and DN4 showed average differences across assessment periods regardless of group (period p < 0.05). ( Table 1 ). 

 The VAS score was higher in the pregabalin 75mg group compared to the placebo group regardless of assessment time point (p = 0.006), and both the VAS and DN4 scores decreased from the preoperative period to subsequent time points, regardless of group (p < 0.001). When the statistical results are analyzed in this way, it is clear that patients showed similar results with a decrease in VAS and an improvement in DN4 at the postoperative assessments regardless of group. In addition, there was no evidence of the occurrence of CRPS in any of the three groups up to 3 months postoperatively ( Table 2 ). 

 The remaining demographic data can be found in Table 3 . 

**Table 1.** Description of pain scores by group during the assessment time points and results of the comparisons.

**Variable/Moment**	**Placebo**	**Group Pregabalin 75mg**	Pregabalin 150mg	**p Group**	**p Moment p Interaction**	
**VAS**				**0.007**	**<0.001**	0.166
**Pre-op.**						
mean ± SD	7.9 ± 2	8.8 ± 1.8	8.4 ± 1.5			
median (p25; p75)	8 (6.3; 10)	10 (8; 10)	9 (7; 10)			
**1 month**						
mean ± SD	1.7 ± 2.4	2.9 ± 3.8	2.1 ± 1.8			
median (p25; p75)	0 (0; 4.8)	0 (0; 6.5)	2 (0; 4)			
**3 months**						
mean ± SD	0.4 ± 0.9	3.5 ± 2.8	0.8 ± 1.5			
median (p25; p75)	0 (0; 0)	3 (1; 6.5)	0 (0; 2)			
**DN4**				0.375	**<0.001**	0.456
**Pre-op.**						
mean ± SD	5.3 ± 2.1	6.2 ± 1.5	6 ± 2.4			
median (p25; p75)	4.5 (4; 7)	7 (5; 7.5)	7 (5; 8)			
**1 month**						
mean ± SD	1.3 ± 1.3	1.5 ± 2	1.8 ± 2			
median (p25; p75)	1 (0; 2)	1 (0; 2.5)	1 (0; 4)			
**3 months**						
mean ± SD	0.6 ± 1.1	1.6 ± 2	0.9 ± 1.3			
median (p25; p75)	0 (0; 1)	1 (0; 3)	0 (0; 2)			

EEG with normal distribution and identity link function, assuming an AR(1) correlation matrix between the time points

**Table 2.** Result of multiple comparisons of pain scores between groups and assessment

		**Mean Difference**	**Standard error**	**Inferior**	**CI (95%)**	
					**Superior**	
VAS Variable Comparison	Placebo - Pregabalina 75mg	-1.74	0.56	0.006	-3.09	-0.39
	Pregabalin Placebo - 150mg	-0.45	0.59	>0.999	-1.86	0.97
	Pregabalin 75mg Pregabalina - 150mg	1.29	0.62	0.110 <0.001	-0.19 5.12	2.78
	Pre-op. - 1 month	6.13	0.42	<0.001	5.62	7.14
	Pre-op. - 3 months	6.76	0.48	0.406	-0.38	7.90
DN4	1 month - 3 months	0.63	0.42	<0.001	1.64	3.66
	Pre-op. - 1 month	4.27	0.26		4.89	
	Pre-op. - 3 months	4.78	0.32	<0.001	4.00	5.55
	1 month - 3 months	0.51	0.26	0.146	-0.11	1.12

**Table 3.** Description of personal and clinical characteristics by group and results of the statistical tests.

**Variable**	Group			Total (N = 40)	^P^
	Placebo (N = 16)	Pregabalin 75mg (N = 13)	Pregabalin 150mg (N = 11)		
**Age(years)**					0.162**
mean ± SD	61 ± 14.8	51.5 ± 8.2	56.2 ± 14.7	56.6 ± 13.3	
median(p25; p75)	60 (47.5; 75.8)	54 (47; 57.5)	52 (41.5; 71.5)	55 (47; 69)	
**Sex**					0.088
Female	15 (93.8)	12 (92.3)	7 (63.6)	34 (85)	
Male	1 (6.3)	1 (7.7)	4 (36.4)	6 (15)	
**Laterality**					**0.031**
Right	7 (43.8)	5 (38.5)	1 (9.1)	13 (32.5)	
Left	4 (25)	5 (38.5)	1 (9.1)	10 (25)	
Bilateral	5 (31.3)	3 (23.1)	9 (81.8)	17 (42.5)	
**Symptom duration (years)**					0.907£
mean ± SD	2.7 ± 2.5	2.6 ± 2.4	2.5 ± 2.5	2.6 ± 2.4	
median (p25; p75)	2 (1; 3)	2 (1.5; 3)	2 (0.7; 3)	2 (1; 3)	

Likelihood ratio test; ** Unpaired Student’s t-test; £ Kruskal-Wallis test

## DISCUSSION

 Pregabalin modulates calcium channels in neurons and has been shown to have antiepileptic and anxiolytic effects as well as analgesic effects in neuropathic pain. ^13^ These results suggested that this substance should be included in the present study to analyze its effect in the postoperative phase of patients with a proven diagnosis of CTS. 

All patients studied were already candidates for surgical treatment of CTS due to failure of clinical treatment or muscle hypotrophy in the thenar region. Although gabapentinoids are approved for the treatment of chronic neuropathic pain, this drug has not yet been shown to be effective in the treatment of postoperative pain in CTS.

 A study by Sadatsun ^14^ found that taking gabapentin, an anticonvulsant with similar effects to pregabalin, in a single dose of 600 mg one hour before induction of anesthesia did not produce significant results in CTS patients. Similar results were found in this study. Even when taking the drug for one month before surgery, only a few patients reported an improvement in symptoms before surgery without avoiding the procedure. 

 However, other studies have shown that gabapentinoids reduced the use of other medications such as opioids in coping with major surgery, ^8^ but this variable was not assessed in this study as patients continued to take analgesics regularly during postoperative follow-up. 

Another aspect to consider in this study is the fact that all patients had a surgical indication before the administration of the medication. Since no statistical difference was found between the control group and the groups with the drug, it is hypothesized that patients with more severe CTS or refractory to conservative treatment did not benefit from the combined treatment of pregabalin and surgery, with the improvement being mainly due to the surgical intervention.

Regarding CRPS, although patients did not manifest this condition during the study period, the literature reports an approximate incidence of 8% in CTS patients. Therefore, a study with a larger patient population could lead to different results.

## CONCLUSION

During the period studied, no significant difference was observed in the use of pregabalin in terms of pain perceived by patients using the VAS and the DN4 and in terms of the occurrence of CRPS.

## References

[1] Chammas M., Boretto J., Burmann L. M., Ramos R. M., Dos Santos F. C., Silva J. B. (2014). Carpal tunnel syndrome – Part I (anatomy, physiology, etiology and diagnosis). Rev Bras Ortop.

[2] Katz J. N., Larson M. G., Sabra A., Kraup C., Stirrat C. R., Sethi R. (1990). The carpal tunnel syndrome: Diagnostic utility of the history and physical examination findings. Ann Intern Med.

[3] Xavier C. R. M., Santos R. D. T. (2001). Carpal tunnel syndrome: treatment using the mini-incision palmar technique. RTO.

[4] Kuschner S. H., Brien W. W., Johnson D., Gellman H. (1991). Complications associated with carpal tunnel release. Orthop Rev.

[5] Cordon F. C. O., Lemonica L. (2002). Complex regional pain syndrome: epidemiology, pathophysiology, clinical manifestations, diagnostic tests and therapeutic proposals. Rev Bras. Anestesiol.

[6] Costa V. V., Oliveira S. B., Fernandes M. C. B., Saraiva R. A. (2011). Incidence of Regional Pain Syndrome after Carpal Tunnel Release. Is there a correlation with anesthesia technique?. Rev Bras Anestesiol.

[7] Zumiotti A. V., Ohno P. E., Prada F. S., Azze R. J. (1996). Complications in the surgical treatment of carpal tunnel syndrome. Rev Bras Ortop.

[8] Clarke H., Pereira S., Kennedy D., Gilron I., Katz J., Gollish J. (2009). Gabapentin decreases morphine consumption and improves functional recovery following total knee arthroplasty. Pain Res Manag.

[9] Tobias A. F. (2019). Randomized, comparative double-blind study on the analgesic effect of pre and postoperative pregabalin in arthroscopic knee ligament correction.

[10] Kirkwood B. R., Sterne J. A. C. (2006). Essential medical statistics.

[11] McCullagh P., Nelder J. A. (1989). Generalized linear models.

[12] Neter J., Kutner M. H., Nachttsheim C. J., Wasserman W. (1996). Applied Linear Statistical Models.

[13] Casas J. D. N. D. S. (2020). The use of anticonvulsants in the perioperative period and its impact on postoperative chronic pain.

[14] Sadatsune E. J., Leal P. D. C., Cossetti R. J. D., Sakata R. K. (2016). Effect of preoperative gabapentin on pain intensity and development of chronic pain after surgical treatment of carpal tunnel syndrome in women: a randomized, double-blind, placebo-controlled trial. São Paulo Med J.

